# Therapeutic role of venous leg compression in worsening heart failure with predominant extravascular congestion: a case report

**DOI:** 10.1093/ehjcr/ytaf343

**Published:** 2025-07-23

**Authors:** Silvia Mínguez, José Civera, Rafael de la Espriella, Diego Iraola, Julio Núñez

**Affiliations:** Cardiology Department, Hospital Clínico Universitario de Valencia, INCLIVA Instituto de Investigación Sanitaria, Avda. Blasco Ibáñez 17, Valencia 46010, Spain; Cardiology Department, Hospital Clínico Universitario de Valencia, INCLIVA Instituto de Investigación Sanitaria, Avda. Blasco Ibáñez 17, Valencia 46010, Spain; Department of Medicine, Universitat de València, Avda. Blasco Ibáñez 13, Valencia 46010, Spain; Cardiology Department, Hospital Clínico Universitario de Valencia, INCLIVA Instituto de Investigación Sanitaria, Avda. Blasco Ibáñez 17, Valencia 46010, Spain; Department of Medicine, Universitat de València, Avda. Blasco Ibáñez 13, Valencia 46010, Spain; Cardiology Department, Hospital Clínico Universitario de Valencia, INCLIVA Instituto de Investigación Sanitaria, Avda. Blasco Ibáñez 17, Valencia 46010, Spain; Cardiology Department, Hospital Clínico Universitario de Valencia, INCLIVA Instituto de Investigación Sanitaria, Avda. Blasco Ibáñez 17, Valencia 46010, Spain; Department of Medicine, Universitat de València, Avda. Blasco Ibáñez 13, Valencia 46010, Spain; Centro de Investigación Biomédica en Red en Enfermedades Cardiovasculares (CIBERCV), Instituto de Salud Carlos III, Avda. Monforte de Lemos, 3-5, Pabellón 11, Planta 0, Madrid 28029, Spain

**Keywords:** Heart failure (HF), Venous leg compression (VLC), Extravascular fluid overload, Case report

## Abstract

**Background:**

Worsening heart failure (WHF) may occasionally present with predominant extravascular fluid overload without clear intravascular congestion. This scenario can challenge traditional diuretic-based strategies, particularly in patients with infiltrative cardiomyopathies such as amyloid light-chain (AL) amyloidosis, where congestion often manifests as volume overload without substantial intravascular volume expansion.

**Case summary:**

This case report highlights the potential efficacy of venous leg compression (VLC)—a therapeutic approach traditionally employed in chronic venous diseases to improve venous return—combined with parenteral diuretic therapy in alleviating signs and symptoms in a 50-year-old woman with AL amyloidosis and WHF. In this patient, VLC was associated with tissue decongestion, enhanced vascular refill, and clinical improvement, suggesting its role in optimizing the diuretic response despite the absence of intravascular congestion.

**Discussion:**

Venous leg compression emerges as a promising adjunctive therapy for managing WHF with predominant extravascular fluid overload, especially when conventional diuretics may be limited by the lack of intravascular congestion. These findings support the potential utility of VLC as a complementary strategy in this subset of patients. Further studies are warranted to establish its safety and efficacy.

Learning points

- Venous leg compression is a therapeutic approach traditionally employed in the management of conditions that involve fluid accumulation at the interstitial compartment.
- In amyloidosis, congestion often manifests as a volume overload without substantial intravascular volume congestion, making conventional diuretic approaches less effective.
- Venous leg compression could have potential utility as a complementary strategy to improve the diuretic response in patients with predominant tissue fluid overload and absence of intravascular congestion.

## Introduction

Venous leg compression (VLC) is a therapeutic approach traditionally employed in the management of conditions such as venous insufficiency and lymphoedema. Recently, its potential role in patients with worsening heart failure (WHF) characterized by predominant extravascular fluid overload has drawn increasing attention. In specific clinical scenarios, WHF may present with significant extravascular fluid overload in the absence of marked intravascular congestion, posing challenges for conventional diuretic-based management strategies.^1,2^ Emerging evidence suggests that VLC may facilitate intravascular refill and enhance diuretic effectiveness in these patients.^3^ However, the application of VLC in WHF remains controversial, and current guidelines provide limited direction regarding its use.^4,5^ Further research is needed to better define the safety and efficacy of this intervention in such complex cases.

## Summary figure

**Figure:**
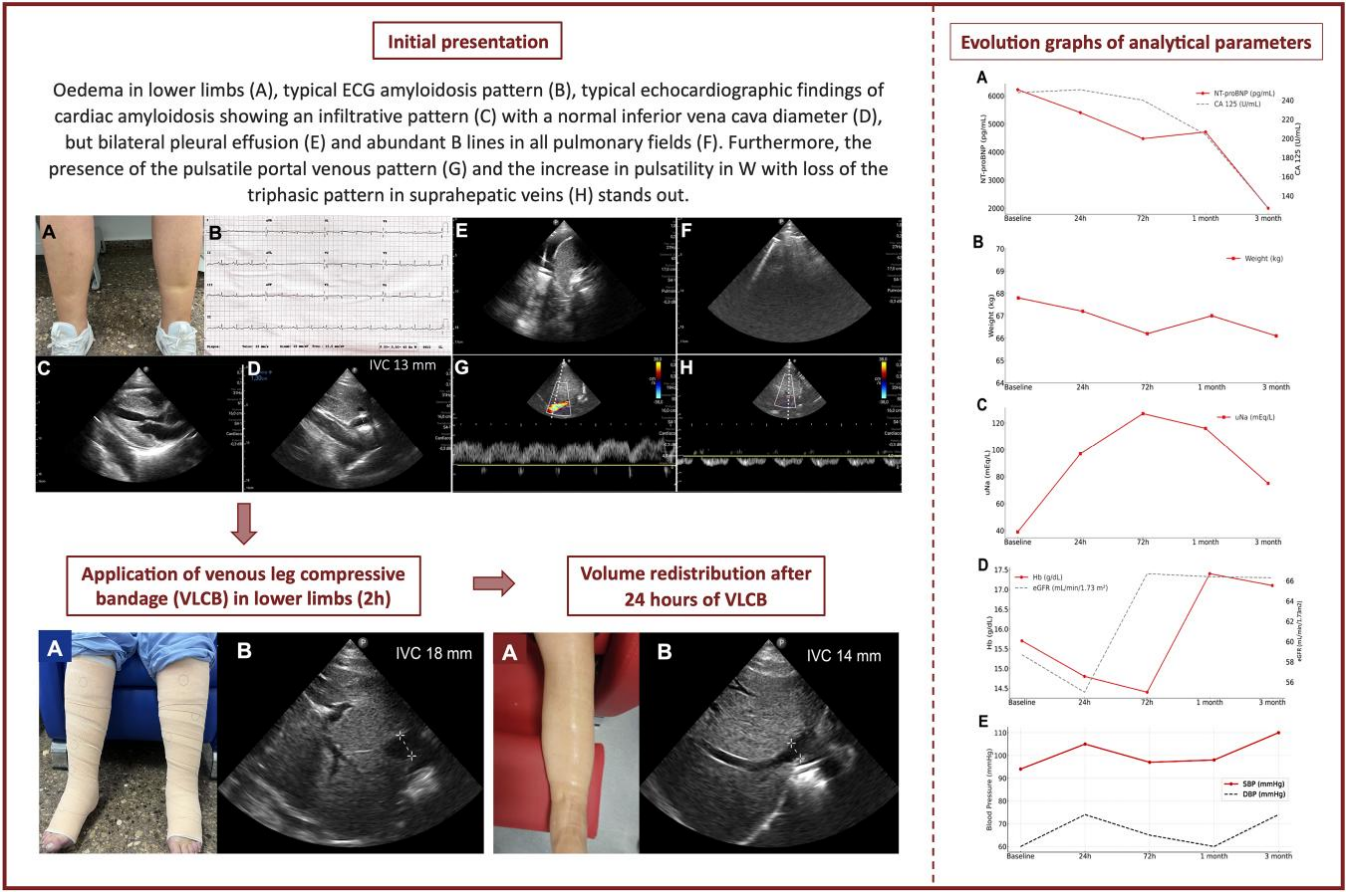


## Case presentation

We report the case of a 50-year-old woman with amyloid light-chain (AL) amyloidosis diagnosed 2 months before the current episode, treated with daratumumab, bortezomib, cyclophosphamide, and dexamethasone. She had a history of complex pulmonary embolism for which she was on anticoagulant therapy, and she was also diagnosed with Lynch syndrome.

During the AL workout, she was diagnosed with heart failure (HF) with preserved ejection fraction, remaining on stable New York Heart Association (NYHA) class I, treated with torsemide 10 mg and eplerenone 50 mg.

**Figure 1:**
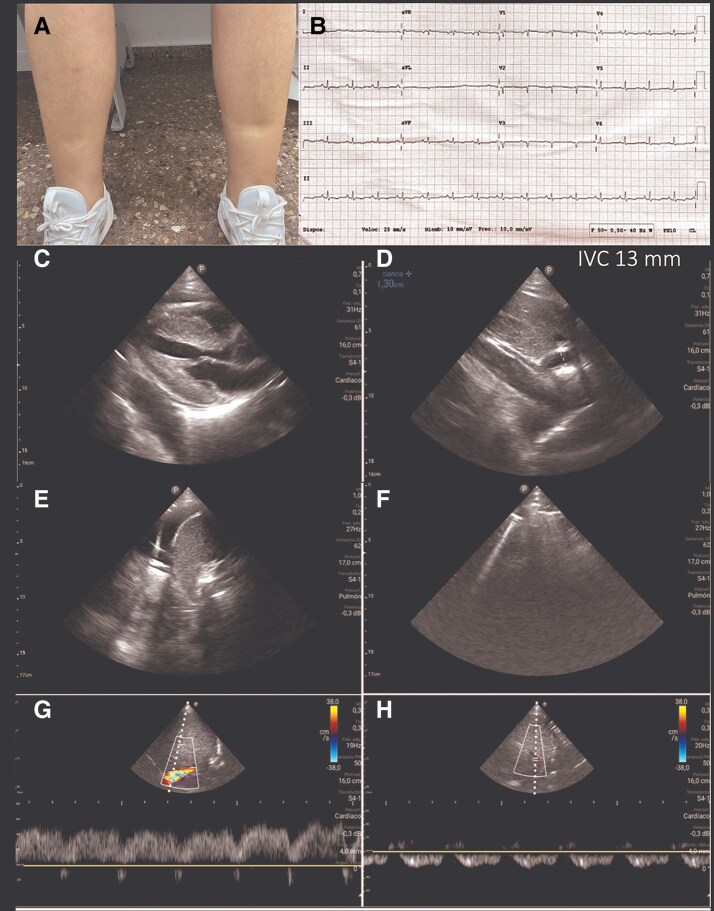
Initial presentation. (*A*) Lower limbs with pedal oedema with pitting III/IV and an ankle circumference of 26 cm. (*B*) Electrocardiogram at presentation. (*C*) Subcostal echocardiographic view: biventricular hypertrophy with amyloid pattern, thickened leaflets, and mild pericardial effusion. (*D*) Inferior vena cava diameter at 13 mm. (*E*) Right pleural effusion in lung ultrasound. (*F*) B lines in lung fields on lung ultrasound. (*G*) Pulsed Doppler in the portal vein showing pulsatility. (*H*) Pulsed Doppler in the suprahepatic vein showing increased W pulsatility and loss of triphasic pattern.

On admission, she presented with worsening effort dyspnoea NYHA class III, palpitations, and peripheral oedema. The blood pressure was 90/60 mmHg and the heart rate was 105 b.p.m. Physical examination showed pedal oedema (pitting III/IV) up to the knees, with an ankle circumference of 26 cm (*Figure 1A*), and no jugular venous distension. Cardiopulmonary auscultation revealed bibasilar crackles.

Regarding complementary tests, the electrocardiogram revealed sinus tachycardia at 105 b.p.m., a narrow QRS with an anterior pseudo-infarction pattern, low voltages in limb leads, and flattened T waves (*Figure 1B*). Transthoracic echocardiography revealed infiltrative involvement of the left ventricle with a preserved left ventricular ejection fraction of 53%. Right ventricular systolic function was mildly reduced, with a TAPSE of 17.5 mm and an S′ velocity of 9.5 cm/s. The left atrium was mildly dilated, with an anteroposterior diameter of 41 mm. Diastolic parameters showed an E wave velocity of 94.3 cm/s, an E/A ratio of 1.1, and a mean E/e′ ratio of 13.6. No significant valvular abnormalities were identified. The inferior vena cava (IVC) measured 13 mm in diameter with inspiratory collapse exceeding 50% (*Figure 1C* and *D*). The ultrasounds also revealed bilateral pleural effusion and abundant B lines in all fields (*Figure 1E* and *F*). Blood laboratory tests indicated an estimated glomerular filtration rate (eGFR) of 58.7 mL/min/1.73m², antigen carbohydrate 125 (CA125) of 248 U/mL, and N-terminal pro B-type natriuretic peptide (NT-proBNP) of 6217 pg/mL. Spot urinary sodium (uNa) was 39 mmol/L. Additionally, the patient showed hypoalbuminemia (2.9 g/dL) and proteinuria in range of nephrotic syndrome (9.83 g/24 h).

**Figure 2:**
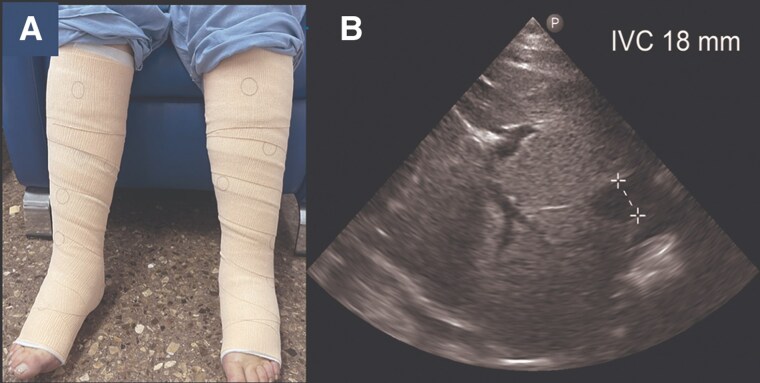
Application of venous leg compression. (*A*) Compression bandage applied to lower limbs. (*B*) Inferior vena cava echocardiography after 2 h of venous leg compression: increase to 18 mm indicating improved intravascular filling.

**Figure 3:**
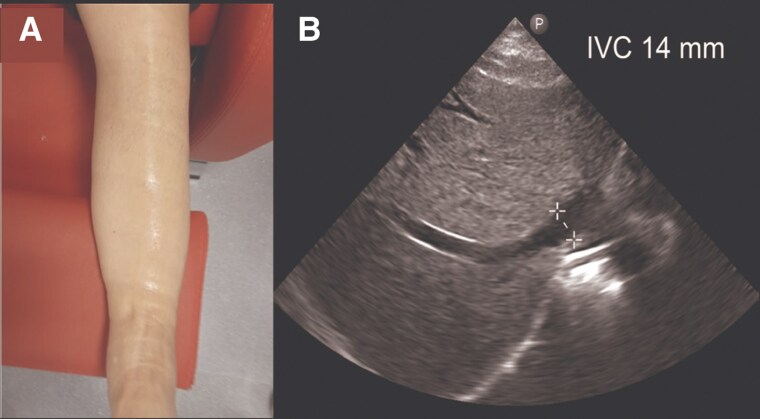
Redistribution of volume after 24 h of venous leg compression. (*A*) Reduced ankle circumference from 26 to 21 cm with residual mild oedema (I/IV). (*B*) Inferior vena cava echocardiography at 14 mm after 24 h of venous leg compression.

**Figure 4:**
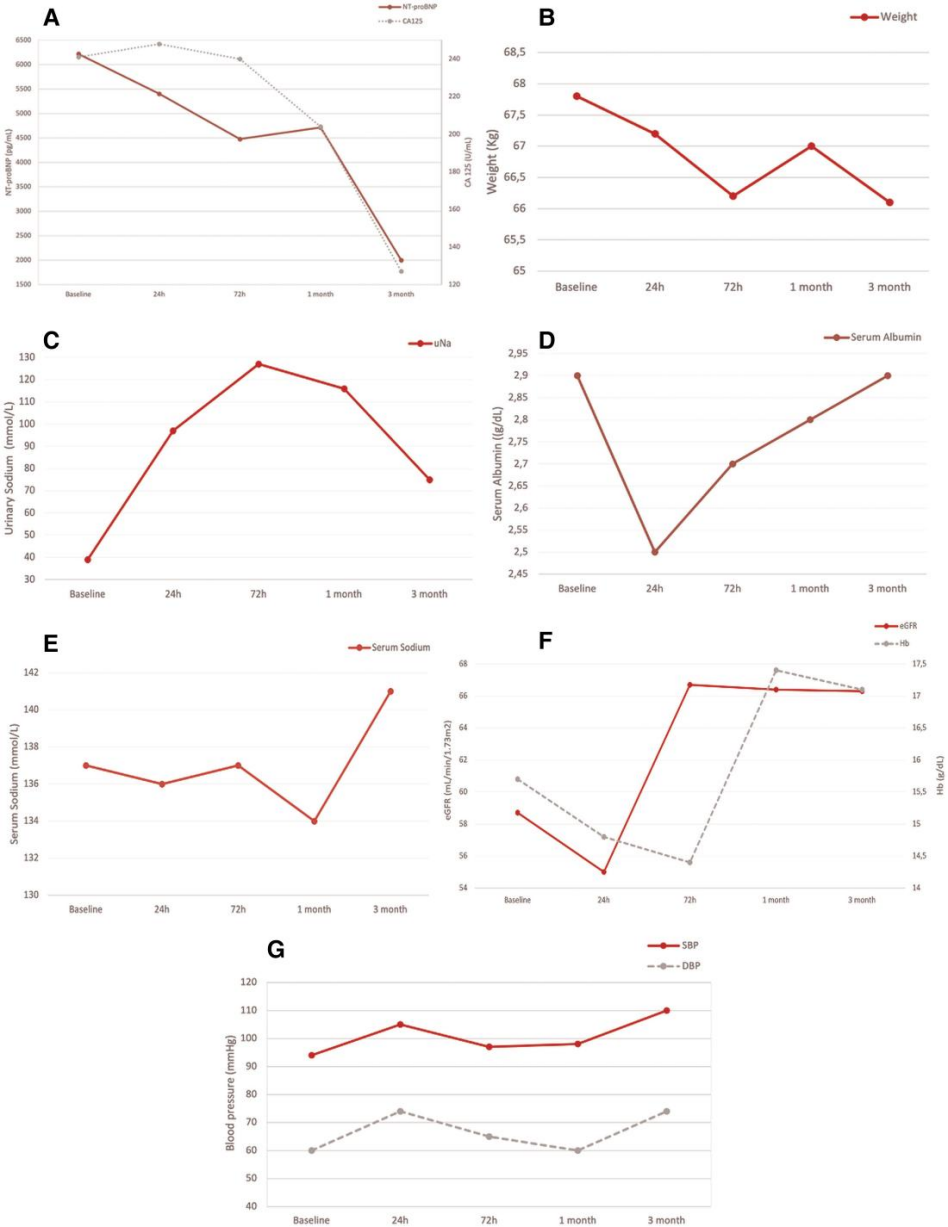
Clinical evolution. (*A*) Trends in congestion markers: N-terminal pro b-type natriuretic peptide and antigen carbohydrate 125. (*B*) Body weight evolution. (*C*) Urinary sodium trajectory. (*D*) Serum albumin trajectory. (*E*) Serum sodium trajectory. (*F*) Changes in haemoglobin and estimated glomerular filtration rate. (*G*) Maintenance of systolic and diastolic blood pressure.

Subcutaneous furosemide (80 mg in 24 h) was initiated along with VLC using elastic bandages (*Figure 2A*). After 2 h, echography revealed an increase in IVC from 13 to 18 mm (*Figure 2B*) together with increased blood pressure (102/63 mmHg) and objectivation of 250 mL of diuresis. After 24 h, clinical improvement was registered. Ankle circumference diameter reduced from 26 to 21 cm, with persisting oedema I/IV (*Figure 3A*). Echography revealed an IVC of 14 mm with inspiratory collapse over 50% (*Figure 3B*). At this moment, subcutaneous furosemide was stopped, and oral furosemide (60 mg/24 h) was started together with eplerenone 50 mg/day, dapagliflozin 10 mg/day, and continuation of VLC. After 48 h, the patient continued showing clinical improvement, so the VLC was removed then, and the oral diuretic strategy was up titrated: furosemide 80 mg/daily, dapagliflozin 10 mg/daily, and eplerenone 50 mg/daily. During subsequent ambulatory visits (up to 3 months), diuretic titration was performed aligned with consistent progressive decongestion evidenced by a drop in NT-proBNP and plasma CA125 levels, weight reduction, and increase in uNa (*Figure 4A–C*). Furthermore, an increase in serum albumin was evident 24 h after the intervention (*Figure 4D*). Trajectories of eGFR, serum sodium, and blood pressure are shown in *Figure 4E–G*, respectively.

## Discussion

This case report highlights VLC’s potential to enhance diuretic response by promoting intravascular refill in WHF patients with peripheral oedemas and absence of systemic intravascular congestion. This congestion pattern is common in advanced heart disease and amyloidosis patients.^1,2,3^ Cardiac amyloidosis often causes volume overload without intravascular congestion, likely due to increased vascular permeability from amyloid fibrils, leading to extravascular fluid overload and intravascular depletion.^2,3^ In this particular setting, hypoalbuminemia could further compromise intravascular volume status, aggravating interstitial fluid accumulation and tissue volume expansion.

Managing interstitial fluid retention is challenging as traditional strategies focus on intravascular volume reduction. The limited tolerability of conventional HF medications complicates management without intravascular congestion.^4^ In this regard, some therapeutic approaches, such as SGLT2 inhibitors, aquaretic, hypertonic saline, or albumin infusions, have been proposed without well-proven utility.^4,5,6^ Likewise, devices to facilitate lymphatic drainage are under investigation.^7^

Venous leg compression, often used for treating oedemas from venous insufficiency and lymphoedema, has uncertain benefits in HF.^7,8^ Some guidelines advise against VLC for HF patients.^8,9^ However, new data suggest its potential safety and utility in patients with WHF, especially those with peripheral oedemas and IVC < 21 mm.^10^ In a study of 20 WHF patients treated with subcutaneous furosemide and VLC for at least 72 h, those with baseline IVC ≤ 21 mm showed a greater increase in 3-h IVC (2.4 vs. 0.8 mm; *P* < 0.001). This increase was linked to better short-term decongestion. A trial to evaluate VLC’s efficacy and safety in WHF patients with IVC ≥ 21 mm is ongoing (*ClinicalTrials.gov Identifier: NCT06418932*).

In this case, VLC in combination with loop diuretic therapy led to marked tissue decongestion, evidenced by a reduction in ankle circumference and pedal oedema, along with improved vascular refill. Furthermore, the observed increase in plasma albumin levels—potentially attributable to haemoconcentration and attenuation of glomerular hyperfiltration—may also be considered contributing factors to the overall clinical improvement. Ongoing monitoring and treatment adjustments were necessary to optimize outcomes. The patient’s response highlights the potential utility of VLC in managing WHF with peripheral oedemas without overt intravascular systemic congestion. Further studies are needed to confirm its efficacy and safety in this clinical scenario.

## Conclusions

Venous leg compression may be a potential option for improving the clinical status and diuretic response in patients with WHF characterized by the presence of peripheral oedemas and lower IVC. Further studies are warranted.

## Data Availability

The data underlying this article are available in the article and its online Supplementary material.

## Lead author biography

Silvia Mínguez is a cardiology intern at the Clinical University Hospital of Valencia, Spain. She completed her medical training also in Valencia, but she is from Vitoria-Gasteiz, a small city placed in the north of the country. Her professional interests focus on clinical cardiology. Outside of medicine, she enjoys staying active by climbing, exploring new cultures through travel, reading contemporary literature, and spending quality time with family and friends.

**Consent:** The authors confirm that written consent for submission and publication of the clinical case including image(s) has been obtained from the patient in line with the COPE guidance.

**Funding:** None declared.
